# Incidence trend of nasopharyngeal carcinoma from 1987 to 2011 in Sihui County, Guangdong Province, South China: an age-period-cohort analysis

**DOI:** 10.1186/s40880-015-0018-6

**Published:** 2015-05-14

**Authors:** Li-Fang Zhang, Yan-Hua Li, Shang-Hang Xie, Wei Ling, Sui-Hong Chen, Qing Liu, Qi-Hong Huang, Su-Mei Cao

**Affiliations:** Department of Cancer Prevention Research, Sun Yat-sen University Cancer Center; State Key Laboratory of Oncology in South China; Collaborative Innovation Center for Cancer Medicine, Guangzhou, 510060 Guangdong P. R. China; School of Public Health, Sun Yat-sen University, Guangzhou, 510080 Guangdong P. R. China; Sihui Cancer Institute, Sihui, 526200 Guangdong P. R. China

**Keywords:** Nasopharyngeal carcinoma, Incidence, Age-Period-Cohort (APC) model, Joinpoint regression, Screening

## Abstract

**Introduction:**

In the past several decades, declining incidences of nasopharyngeal carcinoma (NPC) have been observed in Chinese populations in Hong Kong, Taiwan, Los Angeles, and Singapore. A previous study indicated that the incidence of NPC in Sihui County, South China remained stable until 2002, but whether age, diagnosis period, and birth cohort affect the incidence of NPC remains unknown.

**Methods:**

Age-standardized rates (ASRs) of NPC incidence based on the world standard population were examined in both males and females in Sihui County from 1987 to 2011. Joinpoint regression analysis was conducted to quantify the changes in incidence trends. A Poisson regression age-period-cohort model was used to assess the effects of age, diagnosis period, and birth cohort on the risk of NPC.

**Results:**

The ASRs of NPC incidence during the study period were 30.29/100,000 for males and 13.09/100,000 for females. The incidence of NPC remained stable at a non-significant average annual percent change of 0.2% for males and −1.6% for females throughout the entire period. A significantly increased estimated annual percent change of 6.8% (95% confidence interval, 0.1%–14.0%) was observed from 2003 to 2009 for males. The relative risk of NPC increased with advancing age up to 50–59 and decreased at ages >60 years. The period effect curves on NPC were nearly flat for males and females. The birth cohort effect curve for males showed an increase from the 1922 cohort to the 1957 cohort and a decrease thereafter. In females, there was an undulating increase in the relative risk from the 1922 cohort to the 1972 cohort.

**Conclusions:**

The incidence trends for NPC remained generally stable in Sihui from 1987 to 2011, with an increase from 2003 to 2009. The relative risks of NPC increased in younger females.

## Background

Nasopharyngeal carcinoma (NPC) is a malignant tumor arising from the epithelial cells that cover the surface and line of the nasopharynx. According to the International Agency for Research on Cancer, NPC is rare in most parts of the world, with an incidence of <1/100,000 person-years for both males and females; however, it is a prevalent malignancy in the Cantonese population in South China (including Hong Kong), with an incidence of >20/100,000 in endemic areas [1]. The incidence varies appreciably in different areas of China; the highest risk areas are in South China, especially in Guangdong Province, whereas low rates are generally observed in North China [2,3]. NPC is more predominant in males, with a male-to-female ratio of >2:1 in most populations [4]. Through much extensive research into the etiology of NPC, epidemiologic evidence suggests that Epstein-Barr virus (EBV) infection and genetic susceptibility are important etiologic factors for the disease [5]. Furthermore, diet and environmental factors might also play significant roles in the pathogenesis of NPC, including the consumption of salted fish and other preserved foods that contain nitrosamines, mutagens, and EBV-reactivating substances [6-9], occupational exposure to formaldehyde [6], and cigarette smoking [10,11].

Primary preventative measures against NPC are difficult because the underlying mechanisms of its development are unclear; therefore, screening, as a secondary preventative measure, is the most effective way by which to prevent NPC in endemic areas. Since 1986, several large-scale, population-based screening programs for NPC have been conducted in Sihui County, Guangdong Province, China using EBV-related antibodies as screening markers [12,13]; however, the effect of screening on the incidence and mortality of the disease has not yet been reported.

In the past several decades, declining incidences of NPC have been observed in Chinese populations in Hong Kong, Taiwan, Los Angeles, and Singapore [14-16]. Some important lifestyle factors such as decreased consumption of salted fish and preserved food and increased westernization of dietary habits might be responsible for the observed decrease in incidence; however, the incidence in South China remained stable [2,17,18]. A previous study indicated that the incidence of NPC in Sihui County remained stable until 2002 [17], but whether age, diagnosis period, and birth cohort affect the incidence of NPC remains unknown.

The purpose of our study was to the determine the secular trend of NPC and to explore the potential effects of age, diagnosis period, and birth cohort on the incidence changes of NPC from 1987 to 2011 in Sihui County, South China.

## Methods

### Data sources

The data of the local annual population from 1987 to 2011, excluding the migrant population, were obtained from the Sihui Statistics Department. In 1978, the Sihui Cancer Registry was established to collect NPC incidence data on all residents, and since 1987, a full cancer registry system has been developed to collect all cancer incidence data through the three-grade cancer prevention network. Cancer cases were regularly (approximately once a month) reported by local general practitioners to the central hospital of each town. Health specialists assigned by the Sihui Cancer Research Institute collected the reports, recorded the information onto predesigned cards, and checked the quality of the data. Less than 5.0% of the cancer incidence data of Sihui residents who received treatment in hospitals in other cities were obtained from other cancer registries in Guangdong Province. Linkages were made to the local death register system to supplement underreported data when someone who died of cancer was recorded in the death register system but not in the cancer registry system.

The information collected on the cancer reporting cards included name, sex, birth date, cancer site, diagnosis basis, International Classification of Diseases-10th revision (ICD-10) code, date of diagnosis, cause of death, registered identification number, national identification number, and reporting hospital or medical institution.

### Statistical methods

NPC cases diagnosed between 1987 and 2011 and their corresponding permanent resident populations in Sihui in each single year were stratified by 5-year age intervals. Sex-specific age-standardized rates (ASRs) of incidence were calculated by the direct method using the world standard population method of Segi (1960) [19].

The Joinpoint Regression Program 4.1.1 (National Cancer Institute, Bethesda, MD, USA) was used to fit a series of joined straight lines on a log scale to the trends of annual age-adjusted rates, with the fitted slopes indicating the estimated annual percent change (EAPC) [20]. To quantify the trend over the entire study period, the average annual percent change (AAPC), which is a geometric weighted average of the EAPC trend, was calculated; the weights were equal to the lengths of each segment during a specified fixed interval [21].

Log-linear Poisson regression was performed to investigate the effects of age, diagnosis period, and birth cohort on NPC incidence for each sex. NPC is too infrequent to evaluate before age 30 and after age 75; thus, NPC patients younger than 30 and not younger than 75 were not included in the analysis. NPC cases were categorized into 9 age groups (30–34 to 70–74), 5 period groups (1987–1991 to 2007–2011), and 13 birth cohort groups (1915–1919 to 1975–1979) with a 5-year interval. The birth cohort groups were named by the mid-year of each cohort; for example, the 1915–1919 birth cohort group was named the 1917 cohort. If the variables in the Log-linear Poisson regression were not independent from each other, a non-identifiability problem appeared. To overcome the non-identifiability problem, individual records of cases were used to form a three-way table of age, diagnosis period, and birth cohort [22,23]. Several models—age alone, period alone, cohort alone, age-period, age-cohort, period-cohort, and age-period-cohort (APC)—were generated. The goodness of fit for the specified model was evaluated by the deviance: the closer the deviance was to the degree of freedom, the better the model. The 30–34 age group, the 1987–1991 period group, and the 1942 cohort group were used as the reference groups, given their greater stability compared with other groups. Based on these reference groups, the estimated corresponding relative risks (RRs) could be calculated with the exponentials of regression coefficients of other age, period, and cohort groups; however, because the first and last cohort groups each contained only one datum and the effect estimates were uncertain, the effects for these groups were not presented. The results were considered significant with a two-sided *P* value < 0.05. All statistical analyses were performed using SAS 9.2 (Version 9.2, SAS Institute Inc., Cary, NC, USA).

## Results

### NPC incidence in Sihui County

**Figure 1:**
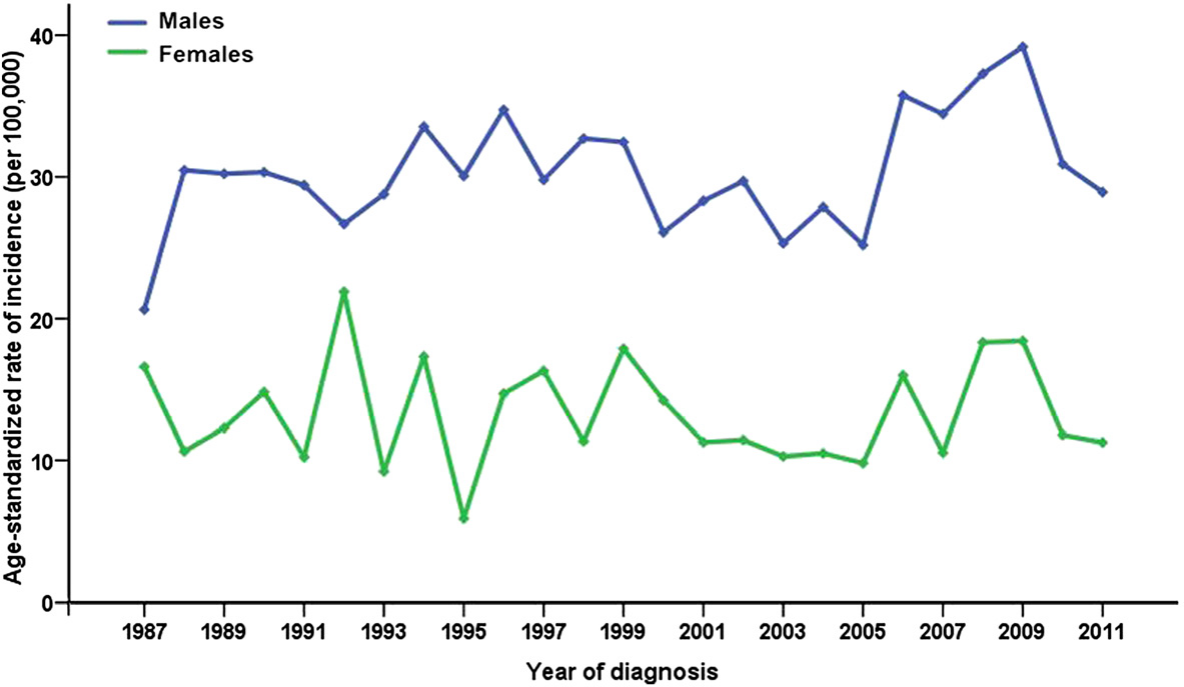
The age-standardized rates (ASRs) of nasopharyngeal carcinoma (NPC) incidence for males and females in Sihui County, Guangdong Province, South China from 1987 to 2011. The ASRs were calculated using the world standard population method of Segi (1960). The NPC incidence in Sihui County fluctuates within a small range for both sexes.

Over the 25-year study period (1987–2011), 2,151 new NPC cases were identified (1,494 males and 657 females) in Sihui County, Guangdong Province, China. Of all these NPC cases newly diagnosed between 1987 and 2011, 85.1% of cases were histologically verified, and only 1.9% had death certificates; the mortality to incidence ratio was 0.641. The crude incidences were 22.37/100,000 for the total population, 30.38/100,000 for males, and 13.99/100,000 for females between 1987 and 2011. The corresponding ASRs were 21.73/100,000 for the total population, 30.29/100,000 for males, and 13.09/100,000 for females. The overall male-to-female ratio of the annual ASR of NPC incidence was 2.3:1 (Table 1). Figure 1 shows that the incidence of NPC in Sihui County between 1988 and 2011 was fluctuated within a small range for both sexes.

**Table 1:** **Age-standardized rates (ASRs) of nasopharyngeal carcinoma (NPC) incidence in Sihui County, Guangdong Province, South China from 1987 to 2011**

**Year**	**Males**			**Females**		
	**Cases**	**Crude incidence (/100,000)**	**ASR (/100,000)**	**Cases**	**Crude incidence (/100,000)**	**ASR (/100,000)**
1987	31	18.15	20.64	25	14.81	16.59
1988	47	27.20	30.47	17	10.00	10.63
1989	49	28.34	30.23	22	12.67	12.27
1990	51	28.49	30.34	26	14.87	14.81
1991	49	27.53	29.42	18	10.20	10.23
1992	48	26.35	26.66	40	22.53	21.90
1993	52	28.07	28.78	19	10.61	9.21
1994	64	33.95	33.56	31	17.19	17.32
1995	58	30.43	30.07	12	6.59	5.89
1996	62	32.04	34.75	28	15.25	14.69
1997	53	27.01	29.79	29	15.66	16.31
1998	56	27.88	32.72	21	11.14	11.34
1999	63	31.25	32.46	29	15.23	17.88
2000	52	25.68	26.07	28	14.65	14.22
2001	59	28.95	28.31	24	12.48	11.28
2002	62	30.33	29.71	23	11.89	11.44
2003	56	27.22	25.30	22	11.33	10.28
2004	58	27.94	27.87	23	11.85	10.50
2005	56	26.60	25.18	21	10.75	9.80
2006	77	36.35	35.77	36	18.22	15.99
2007	71	33.53	34.45	21	10.63	10.54
2008	76	36.03	37.28	38	19.00	18.33
2009	83	39.27	39.18	42	20.79	18.42
2010	83	39.17	30.91	32	15.74	11.79
2011	78	36.52	28.93	30	14.67	11.26

The crude incidence is calculated by dividing the number of annual NPC cases by the total population. The ASRs of incidence were calculated by the direct method using the world standard population method of Segi (1960).

### Change tendency of NPC incidence

**Figure 2:**
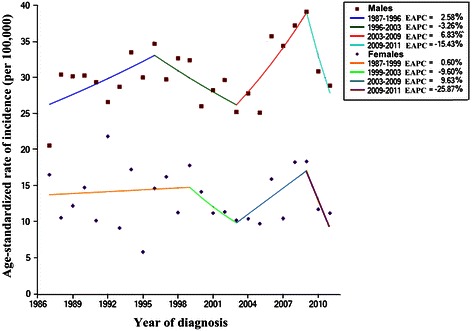
Annual percent changes in NPC incidence stratified by sex in Sihui County, Guangdong Province, South China from 1987 to 2011 with Joinpoint regression. EAPC, estimated annual percent change. ^, *P* < 0.05. From 2003 to 2009, the NPC incidence for males significantly increased, with an EAPC of 6.8% (95% confidence interval [CI], 0.1%–14.0%). The EAPCs during other periods for both sexes were not significant.

Figure 2 depicts the annual percent change in NPC incidence stratified by sex in Sihui County between 1987 and 2011. For males, there was a slight increase (EAPC, 2.6%; 95% confidence interval [CI], −0.6%–5.8%) from 1987 to 1996. From 2003 to 2009, there was a significant increase, with an EAPC of 6.8% (95% CI, 0.1%–14.0%). The EAPC for females was similar to that of males, but no significant changes were observed in different periods. Throughout the entire 25-year period, the incidence of NPC remained stable; the AAPCs were 0.2% for males and −1.6% for females (*P* > 0.05).

### Age-period-cohort effect on NPC incidence

**Figure 3:**
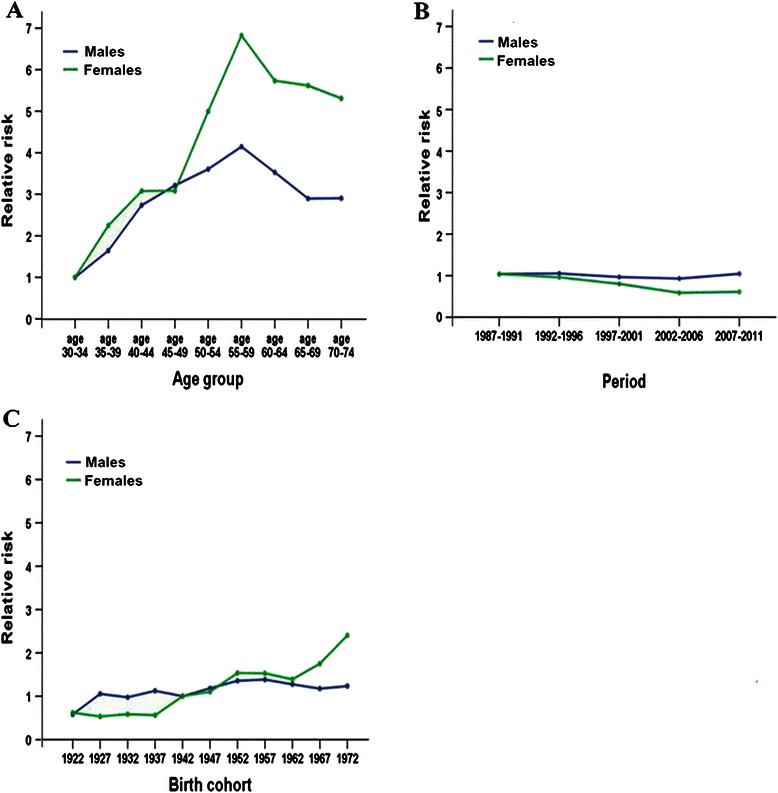
Age, period, and cohort effects obtained from the Poisson age-period-cohort model of NPC in Sihui County, Guangdong Province, South China from 1987 to 2011. **A**, both males and females shared a similar age-effect pattern. The risk of NPC increased with advancing age up to 50–59 years and decreased at ages >60 years. **B**, the curves of period effect on the risk of NPC were virtually flat for both sexes. **C**, the birth cohort curve for males showed that the relative risks of NPC increased from the 1922 cohort to the 1957 cohort and decreased after the 1957 cohort. For females, the relative risks increased with undulation from the 1922 cohort to the 1972 cohort and peaked near the 1972 cohort.

Table 2 shows that the APC model fit better than other models for both males and females because the deviance in this model was the closest to the degree of freedom. The effects of age, diagnosis period, and birth cohort on the risk of NPC for males and females are illustrated in Table 3. Both males and females shared a similar age-effect pattern. The risk of NPC increased with advancing age up to 50–59 and decreased at ages >60 years (Figure 3A). The curves of period effect on the risk of NPC were virtually flat for both sexes (Figure 3B). The curve for males by birth cohort showed that the RRs increased from the 1922 cohort to the 1957 cohort and decreased after the 1957 cohort; for females, the RRs increased with undulation from the 1922 cohort to the 1972 cohort and peaked near the 1972 cohort (Figure 3C).

**Table 2:** **The results of Poisson regression age-period-cohort models for NPC incidence in Sihui County, Guangdong Province, South China from 1987 to 2011**

**Variable**	**Sex**	**DF**	**Deviance**	**Deviance/DF**
Age	Male	36	50.38	1.40
	Female	36	42.67	1.19
Period	Male	40	290.03	7.25
	Female	40	114.45	2.86
Cohort	Male	32	151.03	4.72
	Female	32	57.77	1.80
Age-period	Male	32	46.36	1.45
	Female	32	39.89	1.25
Age-cohort	Male	24	31.97	1.33
	Female	24	23.50	0.98
Period-cohort	Male	28	100.57	3.59
	Female	28	46.23	1.65
Age-period-cohort	Male	21	28.70	1.37
	Female	21	20.55	0.98

DF, degree of freedom.

**Table 3:** **Relative risk (RR) and 95% confidence interval (CI) of NPC for males and females by age-period-cohort analysis in Sihui County, Guangdong Province, South China**

**Parameter**	**Category**	**Males**		**Females**	
		**RR**	**95% CI**	**RR**	**95% CI**
Age (years)	30-34	1.00 (Ref)		1.00 (Ref)	
	35-39	1.64	1.24–2.20	2.25	1.59–3.23
	40-44	2.73	2.05–3.68	3.08	2.07–4.71
	45-49	3.21	2.36–4.45	3.08	1.93–5.23
	50-54	3.60	2.53–5.28	5.00	2.93–9.48
	55-59	4.15	2.75–6.51	6.82	3.65–14.77
	60-64	3.52	2.16–6.04	5.74	2.68–14.57
	65-69	2.89	1.60–5.49	5.62	2.31–16.65
	70-74	2.90	1.41–6.26	5.31	2.55–15.00
Period	1987-1991	1.00 (Ref)		1.00 (Ref)	
	1992-1996	1.01	0.80–1.28	0.92	0.68–1.26
	1997-2001	0.93	0.70–1.23	0.77	0.51–1.14
	2002-2006	0.89	0.63–1.25	0.56	0.33–0.92
	2007-2011	1.01	0.68–1.45	0.59	0.31–1.01
Cohort	1920-1924	0.58	0.26–1.24	0.62	0.25–1.45
	1925-1929	1.05	0.62–1.77	0.53	0.26–1.06
	1930-1934	0.97	0.64–1.47	0.59	0.34–1.00
	1935-1939	1.12	0.82–1.55	0.56	0.36–0.87
	1940-1944	1.00 (Ref)		1.00 (Ref)	
	1945-1949	1.18	0.91–1.54	1.10	0.77–1.58
	1950-1954	1.36	1.03–1.79	1.54	1.06–2.30
	1955-1959	1.38	1.02–1.91	1.53	0.97–2.56
	1960-1964	1.27	0.88–1.89	1.39	0.79–2.69
	1965-1969	1.18	0.75–1.89	1.75	0.90–3.89
	1970-1974	1.23	0.73–2.17	2.40	1.11–6.14

## Discussion

The present study demonstrated that the secular trend of NPC incidence remained stable with only slight fluctuation in Sihui County from 1987 to 2011. A similar incidence trend was observed in other regions of Guangdong and Guangxi Provinces, China [2,17]; however, the incidence of NPC has declined steadily in Hong Kong and Taiwan over recent decades [14,24]. The migrant epidemiologic studies conducted in Chinese populations in Singapore, Hong Kong, and Los Angeles showed a rapid reduction in NPC incidence from 1973 to 1997 in both males and females. In Singapore, the incidence of NPC in Chinese populations dropped from 1993 to 1997 in both sexes [15]. The phenomenon of heterogeneous secular trends of NPC incidence among different areas and populations might be partially explained by the diversity of environmental risk factors in different areas.

Some important lifestyle factors for NPC, such as the consumption of salted fish and preserved food and were expected to decrease as environmental changes and the westernization of dietary habits became more prevalent. This is presumed to be the reason for the decline in NPC incidence in Hong Kong and Taiwan [14,24]. During recent decades, with the rapid upward trend in the economy of Sihui County, changing lifestyles, dietary habits, and living conditions were also remarkable; however, we observed that the secular trend in NPC incidence remained stable until 2011. This coincided with the findings of other researchers [2,17,18]. We presumed that this trend might be because the effects of the changes in environmental factors lag behind the incidence of NPC or because some unknown environmental factors still exist in South China.

Our Joinpoint regression data suggested that the incidence of NPC increased from 1987 to 1999 for females and from 1987 to 1996 for males. Another significant increase for males appeared in 2003–2009, but each increase was followed by a decrease. These fluctuations within different periods are partly because of the two population-based NPC screening programs conducted in Sihui County: the first recruited participants from four towns in 1987, 1992 until 1997 [12]; the second was an ongoing screening project, of which the first round was conducted in six towns from 2008 to 2009 [13]. More early-stage NPC cases were detected, which indicated that the incidence increased during the screening period.

The age effect reflects physiologic differences among different age groups in the susceptibility to a given disease. In the present study, we found that the risk of NPC increased with age until 55-59 years and decreased thereafter; this finding was consistent with data from previous studies in endemic areas [25]. The period effect usually reflects the roles of multiple factors on the given disease that equally affect all age groups during a period of time. In our study, the introduction of new diagnostic techniques, changes in environmental factors, and modulation of lifestyles together might result in the unchanged effect by period. The birth cohort effect on NPC might result from different environmental exposure levels in different birth cohorts. The effect of lifestyle factors, which might be fixed early in life, is most likely reflected by the cohort effect [24]. Birth cohort-stratified incidences in males and females showed two trends. In males, the cohort effect is curved, with NPC risks increasing from the earliest cohorts, peaking in the 1957 cohort, and decreasing in younger cohorts from the 1957 cohort to the 1972 cohort; however, the RRs increased with undulation in the 1972 cohort of females. We presumed that the increased NPC risk in the younger female cohort might be due to increased cigarette smoking. A meta-analysis reported that cigarette smoking increased the risk of NPC [11]. Evidence from several studies conducted in Guangdong Province showed that the smoking rate was higher in males than in females but the smoking rate in males has decreased, whereas that in females has increased in recent years [26,27]. Moreover, exposure to secondhand smoke was more prevalent in females than in males [26,27].

There are several limitations that must be considered when interpreting the results from this study. With respect to the statistical methods, when using the APC model analysis, the sample size appeared too small to achieve robust estimates; thus, the age, diagnosis period, and birth cohort effects should be interpreted with caution. In addition, our study lacked adequate data to determine the incidence trend by histological subtype because the detailed histological subtype data for each cancer case were not included on the cancer report card in Sihui County. In the future, it might be better to analyze the incidence trend by different histological subtypes of NPC if the relevant data can be collected.

## Conclusions

In conclusion, the incidence trends for NPC remained generally stable in Sihui County from 1987 to 2011, with an increase during 2003–2009. The RRs of NPC in younger female birth cohorts increased; the underlying reasons for this will be further explored. Because NPC remains a significant health burden in Sihui County, it is worthwhile to conduct epidemiologic studies on genetic susceptibility, environmental factors, and their interactions.
